# Regulation of Myogenic Activity by Substrate and Electrical Stimulation *In Vitro*

**DOI:** 10.1089/biores.2019.0016

**Published:** 2019-07-30

**Authors:** Anjali Patel, Sara Vendrell-Gonzalez, Gabriel Haas, Madison Marcinczyk, Natalia Ziemkiewicz, Muhamed Talovic, Jonathan S. Fisher, Koyal Garg

**Affiliations:** ^1^ Department of Biomedical Engineering, Parks College of Engineering, Aviation, and Technology, Saint Louis University, St. Louis, Missouri.; ^2^ Department of Biology, College of Arts and Sciences, Saint Louis University, St. Louis, Missouri.

**Keywords:** collagen, electrical stimulation, laminin, myoblasts

## Abstract

Skeletal muscle has a remarkable regenerative capacity in response to mild injury. However, when muscle is severely injured, muscle regeneration is impaired due to the loss of muscle-resident stem cells, known as satellite cells. Fibrotic tissue, primarily comprising collagen I (COL), is deposited with this critical loss of muscle. In recent studies, supplementation of laminin (LM)-111 has been shown to improve skeletal muscle regeneration in several models of disease and injury. Additionally, electrical stimulation (E-stim) has been investigated as a possible rehabilitation therapy to improve muscle's functional recovery. This study investigated the role of E-stim and substrate in regulating myogenic response. C2C12 myoblasts were allowed to differentiate into myotubes on COL- and LM-coated polydimethylsiloxane molds. The myotubes were subjected to E-stim and compared with nonstimulated controls. While E-stim resulted in increased myogenic activity, irrespective of substrate, LM supported increased proliferation and uniform distribution of C2C12 myoblasts. In addition, C2C12 myoblasts cultured on LM showed higher Sirtuin 1, mammalian target of rapamycin, desmin, nitric oxide, and vascular endothelial growth factor expression. Taken together, these results suggest that an LM substrate is more conducive to myoblast growth and differentiation in response to E-stim *in vitro*.

## Introduction

The extracellular matrix (ECM) in skeletal muscle plays a vital role in force transmission as well as development, maintenance, and regulation of the stem cell niche.^1^ An important component of the muscle ECM is collagen I (COL), the primary isoform of collagen, which is found in the perimysium.^2^ The COL triple helix is a heterotrimeric protein with two identical α1(I) chains and one α2(I) chain and it usually imparts tensile strength in the ECM.^3^ Pathological overexpression of collagen results in muscle fibrosis, which is known to hinder muscle regeneration in several models of disease, injury, and aging.^1,4–7^ Fibrotic tissue deposition is known to impair muscle regeneration by dysregulating proliferation and differentiation of satellite cells^4,8^ as well as other muscle-resident stem cells.^8,9^

Laminin (LM) is a heterotrimeric glycoprotein with three chains (α, β, and γ) that is found in the basal lamina of the satellite cell niche. A particular LM isoform, LM-111 (LM), is expressed in the epithelium and endothelium primarily during fetal and embryonic development and is absent in adult tissues.^10^ Studies have shown that LM supplementation dramatically improves regenerative capacity in several models of muscular disease^11–13^ and injury^14^ by increasing satellite cell activity. *In vitro*, LM has been shown to better promote muscle stem cell proliferation and differentiation in comparison with other ECM components such as COL or gelatin.^15,16^ LM also increases acetylcholine receptor clustering, a critical step in the formation of neuromuscular junctions necessary for muscle innervation.^17^

Since the musculoskeletal system is highly adaptive to increased use,^2^ physical therapy is being widely used to promote muscle repair, improve function, and maintain muscle mass.^18^ Electrical stimulation (E-stim) has been used as a rehabilitation therapy method and substitutes for voluntary physical exercise.^19^ Some studies have found that direct E-stim to denervated muscles increases muscle mass and average fiber diameter.^20,21^ Additionally, E-stim has been used on elderly patients to counteract neuromuscular disability, strengthen muscles, and maintain muscle mass.^22^ However, the effects of E-stim on muscle atrophy are dependent on the type of disuse model and various stimulation conditions such as intensity, frequency, and number of contractions.^23–25^ It has also been reported that E-stim in the presence of fibrotic tissue fails to promote nerve regeneration, hindering its effectiveness as a rehabilitation therapy.^26^

E-stim is also being studied as an *in vitro* exercise model that mimics *in vivo* muscle adaptations. It has been shown that 24 h after E-stim, myotubes increase expression of myokines and markers associated with mitochondrial biogenesis in differentiated myotubes.^27–29^ The immediate effects of E-stim *in vivo* include activation of glucose uptake and glycogenolysis.^30^ Adaptations of metabolic properties in skeletal muscle after exercise are reflected by increased mitochondrial content and improved oxidative capacity.^31,32^ During skeletal myotube development, myoblasts first upregulate primary transcription factors such as myoblast determination protein 1 (MyoD) and then overexpress secondary myogenic factors such as myogenin.^33^ In addition, the timing of E-stim is critical in myoblast differentiation.^34^ If stimulation is applied before myotube formation, the myoblasts are unable to benefit from stimulation, producing myotubes with poor sarcomeric structure. However, if E-stim is applied after myotubes are assembled, beneficial results of myogenesis are seen, such as a higher level of striated myotubes and contractile protein expression.^34^

The goal of this study was to characterize the C2C12 myoblast response to E-stim in the context of the substrate. We hypothesized that E-stim in the presence of LM would support the expression of factors that enhance skeletal muscle regeneration, while culture on COL would impede this response.

## Materials and Methods

### Preparation of LM- and collagen-coated molds

Polydimethylsiloxane (PDMS) molds (4 cm^2^) were autoclaved before use. Rat tail collagen (COL) I (Gibco, 9.33 mg/mL) and LM-111 (Trevigen, 6 mg/mL) solution was diluted to 200 μg/mL using 1× phosphate-buffered saline (PBS). A total of 500 μL of the diluted COL and LM-111 solution was added to PDMS molds. The molds were incubated at 37°C for 1 h, after which the collagen and LM solutions were removed and rinsed in 1 × PBS before cell seeding. PDMS molds were used with the C-Pace E-stim system as per manufacturer's instructions. PDMS is typically chosen for such studies due to its (1) biocompatibility, (2) transparency for visualization of cells under a microscope, and (3) high adsorption capacity that permits coating with various ECM proteins such as collagen and LM.^35^

### C2C12 myoblast cell culture

C2C12 myoblasts were seeded on coated molds at a density of 400,000 cells/mold in Dulbecco's Modified Eagle Medium (DMEM)-F12 media containing 10% fetal bovine serum and 1% penicillin–streptomycin (P/S). Once confluent, the cells were switched to differentiation media, DMEM-F12 media containing 2% horse serum and 1% P/S, to induce myotube formation. We chose a cell density of 400,000 cells/mold to account for the large area of the PDMS molds (4 cm^2^) and potential cell death due to serum starvation and E-stim.

### Electrical stimulation

The cell-seeded COL and LM-111 molds either received no stimulation (NS) or E-stim for 1 h per day for 3 days (*n* = 3/group/time point). E-stim was applied using a multichannel culture stimulator (IonOptix C-Pace EM). Samples were exposed to a 1 V bipolar rectangular pulse sequence of 2-ms duration and 2-Hz frequency. The resulting electrical field intensity was 50 V/m. Cell culture supernatants were collected 1 h after stimulation on days 1 and 3.

### Cellular morphology

Cellular attachment and morphology on the molds were analyzed on day 3 by fixing cell-seeded molds in 4% paraformaldehyde and immunostaining with desmin (Abcam), MyoD (Thermo Fisher Scientific), and DAPI (Vector Laboratories). Images were captured at 20× magnification using a Zeiss Axiocam fluorescence microscope. Cellular alignment/orientation was measured using ImageJ using the fast Fourier transform (FFT) method, as described previously.^36^ Pixel intensities were plotted between 0° and 360°, and the degree of alignment in the FFT data was reflected by sharpness and height of the peak shown on the plot.

### Quantification of cell-secreted factors

Production of vascular endothelial growth factor (VEGF) and interleukin-6 (IL-6) by C2C12 myoblasts was quantified in cell culture supernatants using ELISA (PeproTech), as per manufacturer's instructions. Production of nitric oxide (NO) by C2C12 myoblasts was quantified in cell culture supernatants using a colorimetric kit (Abcam), as per manufacturer's instructions.

### Western blotting

Protein lysates from myoblasts collected on days 1 and 3 were probed for Sirtuin 1 (SIRT1) (Cell Signaling Technology), mammalian target of rapamycin (mTOR) (Cell Signaling Technology), desmin (Abcam), myogenin (Millipore), α-actinin (Cell Signaling Technology), MyoD (Thermo Fisher Scientific), atrogin-1 (FBXO32; Invitrogen), and glyceraldehyde 3-phosphate dehydrogenase (Millipore) by western blot. Protein lysates were resolved by SDS-PAGE using total protein from cell lysates (30 μg) on 4–20% Tris-glycine gels (Bio-Rad). Equal protein loading was confirmed by Ponceau staining. The western blot was performed as previously described.^6,37^

### XTT cell proliferation assay

The XTT cell proliferation assay (Sigma-Aldrich) was performed to determine the total number of cells on the molds on days 1 and 3. The XTT labeling solution, comprising the XTT labeling agent and electron-coupling reagent, was added directly to the molds. After incubation at 37°C for 3 h, the XTT labeling solution was transferred to a 96-well plate. A standard curve was generated using cultured myoblasts. Absorbance of the samples was read at 475 nm on a microplate reader.

### Statistical analysis

All data are presented as mean ± standard error of the mean. Data were analyzed and graphed using GraphPad Prism 6. A two-way analysis of variance was performed to determine whether a significant interaction or main effect existed between factors for each dependent variable under consideration. When appropriate, a least significant difference *post hoc* comparison was performed to identify the source of significance with *p* ≤ 0.05.

## Results

### Regulation of C2C12 morphology by substrate and stimulation

**FIG. 1.**
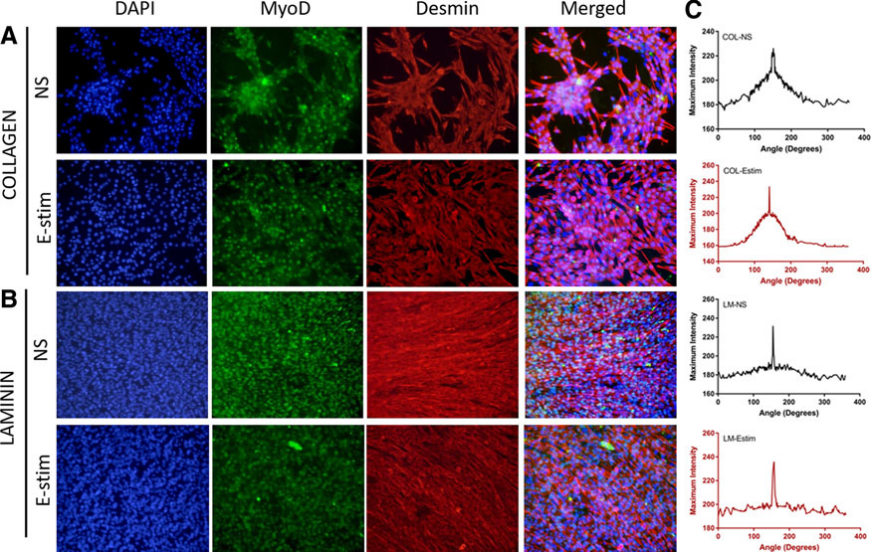
C2C12 myoblasts cultured on COL and LM (*n* = 3/group) are responsive to external stimuli. Cells were cultured under the conditions of NS and E-stim for 3 days. **(A)** Aggregate-like structures are formed by C2C12 myoblasts cultured on COL. **(B)** Desmin^+^ MyoD^+^ myotube formation is promoted by C2C12 myoblasts cultured on LM. **(C)** FFT analysis of histological images taken to analyze myotube alignment. COL, collagen I; E-stim, electrical stimulation; FFT, fast Fourier transform; LM, laminin; MyoD, myoblast determination protein 1; NS, no stimulation.

C2C12 myoblasts cultured on collagen type I (COL) formed multicell aggregates under both NS and E-stim conditions (Fig. 1A). In contrast, myotubes were uniformly distributed on LM-111-coated PDMS molds under both NS and E-stim conditions (Fig. 1B). These results suggest that cellular organization is different on COL and LM substrates. While COL promotes cellular aggregation, LM promotes myoblast spreading and elongation. The cellular alignment and orientation quantification results are also presented in Figure 1. For both the stimulated and unstimulated groups, the LM substrate showed sharper peaks compared with COL, suggesting greater cellular alignment (Fig. 1C).

### Metabolic and myogenic activity of C2C12 myoblasts in response to substrate and stimulation

**FIG. 2.**
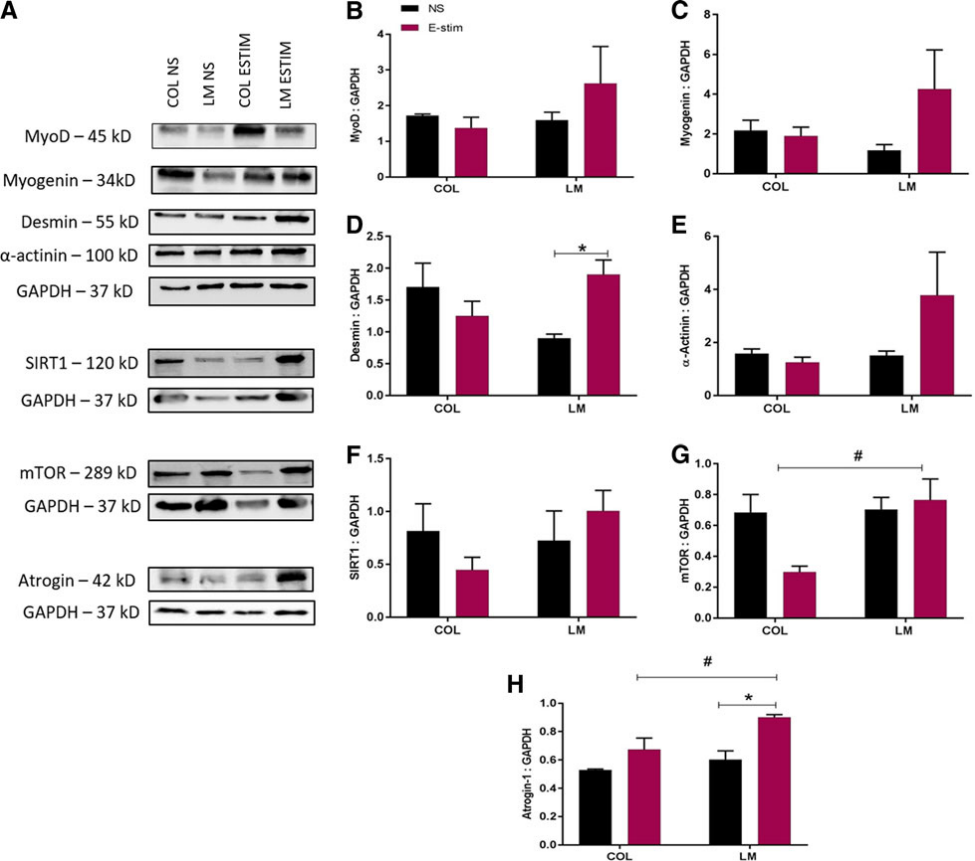
Quantification of myogenic and metabolic markers on day 1 in C2C12 myoblasts cultured on COL and LM (*n* = 3/group) exposed to E-stim and NS conditions. **(A)** Representative bands for MyoD, myogenin, desmin, α-actinin, SIRT1, mTOR, atrogin-1, and GAPDH are shown. Quantification of band intensity for **(B)** MyoD, **(C)** myogenin, **(D)** desmin, **(E)** α-actinin, **(F)** SIRT1, **(G)** mTOR, and **(H)** atrogin-1 is shown. Statistical significance between NS and E-stim conditions (*p < 0.05) is denoted by an asterisk, while that between COL and LM (*p < 0.05) is denoted by a hashtag. GAPDH, glyceraldehyde 3-phosphate dehydrogenase; mTOR, mammalian target of rapamycin; SIRT1, Sirtuin 1.

**FIG. 3.**
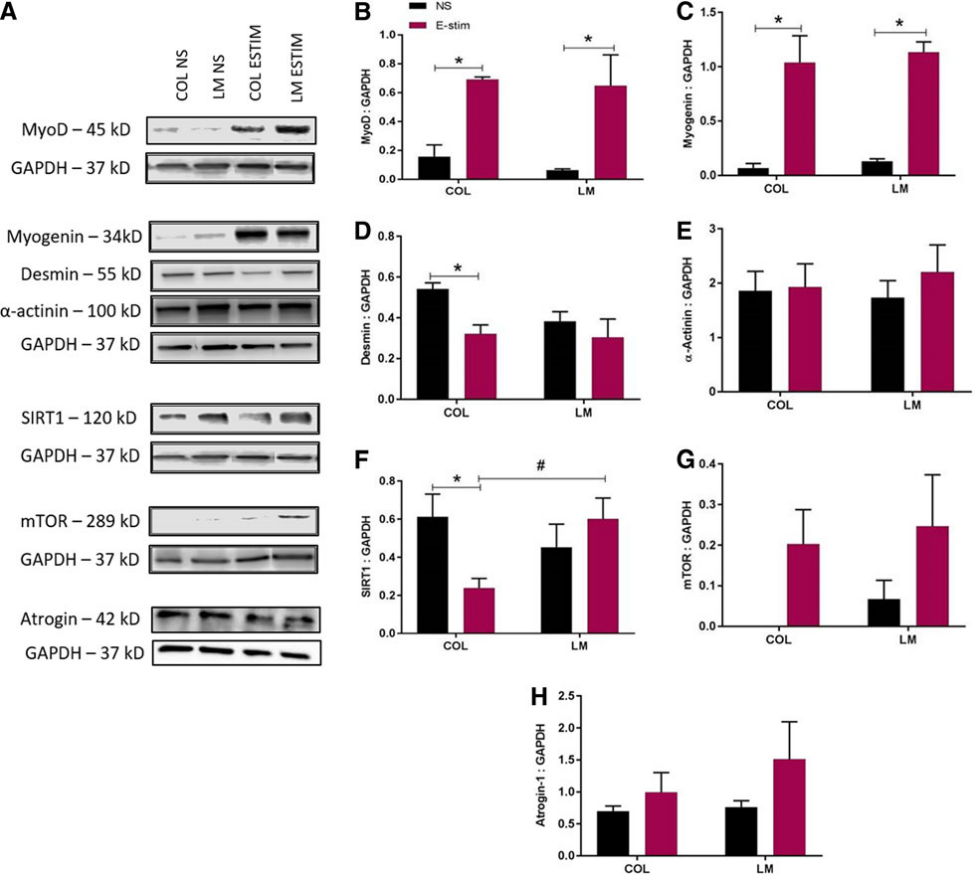
Quantification of myogenic and metabolic markers on day 3 in C2C12 myoblasts cultured on COL and LM (*n* = 3/group) exposed to E-stim and NS conditions. **(A)** Representative bands for MyoD, myogenin, desmin, α-actinin, SIRT1, mTOR, atrogin-1, and GAPDH are shown. Quantification of band intensity for **(B)** MyoD, **(C)** myogenin, **(D)** desmin, **(E)** α-actinin, **(F)** SIRT1, **(G)** mTOR, and **(H)** atrogin-1 is shown. Statistical significance between NS and E-stim conditions (*p < 0.05) is denoted by an asterisk, while that between COL and LM (*p < 0.05) is denoted by a number sign.

The expression of MyoD, myogenin, desmin, α-actinin, SIRT1, mTOR, and atrogin-1 was quantified in the myoblast lysates on days 1 and 3 of culture (Figs. 2A–H and 3A–H). On day 1, a significant increase in desmin expression (Fig. 2D) was found in electrically stimulated myoblasts cultured on LM compared with unstimulated controls (*p* = 0.0195). Additionally, on day 1, a significant increase in mTOR expression (Fig. 2G) was found in electrically stimulated myoblasts cultured on LM compared with those cultured on COL (*p* = 0.0104). The protein expression of atrogin-1 (Fig. 2H) was significantly higher in electrically stimulated myoblasts cultured on LM compared with those cultured on COL (*p* = 0.0148). On the LM substrate, expression of atrogin-1 was also higher on electrically stimulated myoblasts compared with unstimulated controls (*p* = 0.0036).

On day 3, a significant increase in MyoD expression (Fig. 3B) was found in electrically stimulated myoblasts cultured on both COL (*p* = 0.0111) and LM (*p* = 0.0071) compared with unstimulated controls. A significant increase in myogenin (Fig. 3C) was also observed on day 3 in electrically stimulated myoblasts cultured on both COL (*p* = 0.0009) and LM (*p* = 0.0007) compared with unstimulated controls.

Compared with unstimulated controls, myoblasts cultured on COL showed a significant decrease in desmin (Fig. 3D, *p* = 0.0265) and SIRT1 (Fig. 3F, *p* = 0.0346) expression in response to E-stim on day 3. A significant increase in SIRT1 expression was observed in electrically stimulated myoblasts cultured on LM (*p* = 0.0386) in comparison with those cultured on COL.

### Cellular growth and trophic factor secretion

**FIG. 4.**
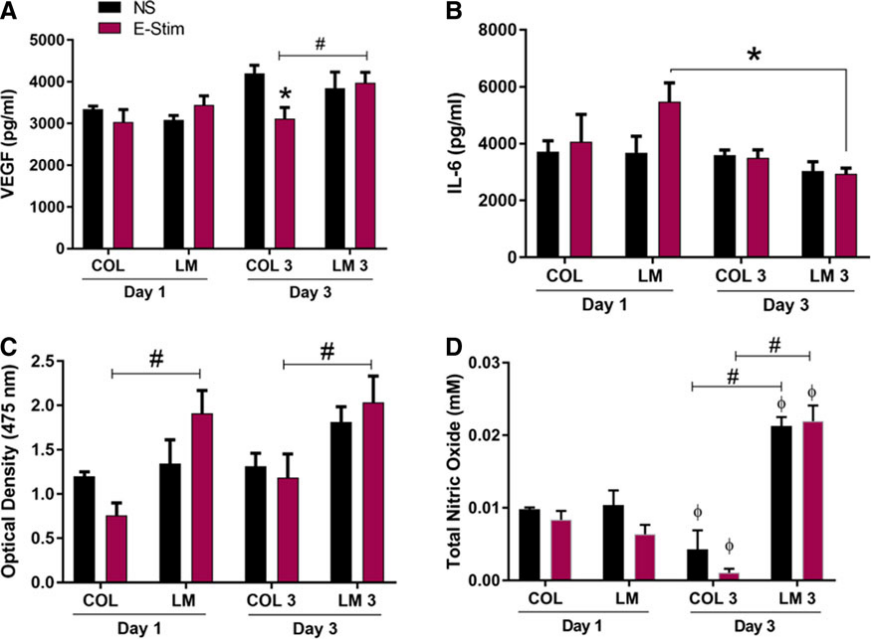
E-stim supports myoblast growth and myokine secretion on the LM substrate. Quantification of **(A)** VEGF and **(B)** IL-6 from cell culture supernatants (*n* = 3/group). **(C)** Relative quantity of metabolically active cells measured by the XTT cellular proliferation assay (*n* = 3/group). **(D)** Total nitric oxide produced by unstimulated and electrically stimulated myoblasts on days 1 and 3. Statistical significance between NS and E-stim conditions (*p* < 0.05) is denoted by an asterisk, while that between COL and LM (*p* < 0.05) is denoted by a number sign. Statistical differences between days 1 and 3 are denoted by the phi sign (φ). IL-6, interleukin-6; VEGF, vascular endothelial growth factor.

The levels of VEGF and IL-6 were quantified in cell culture supernatants on days 1 and 3 (Fig. 4A, B). VEGF is a proregenerative growth factor that plays a role in myoblast migration and survival.^38,39^ The amount of VEGF produced on day 1 was similar across all groups. On day 3, production of VEGF was significantly decreased on COL E-stim in comparison with COL NS (*p* = 0.0062) and maintained on LM for both NS and E-stim conditions. Expression was also significantly increased on LM E-stim in comparison with COL E-stim (*p* = 0.0246).

IL-6 is a proinflammatory cytokine that is produced in response to muscle damage and exercise.^40,41^ On day 1, levels of IL-6 production were relatively stable with a trend for higher production on LM E-stim. On day 3, levels of IL-6 production were significantly decreased for LM E-stim in comparison with day 1 (*p* = 0.0031). These results indicate that by day 3, the myotubes have acclimated to stimulation conditions and IL-6 levels have returned to baseline.

The XTT cellular proliferation assay (Fig. 4C) showed that under E-stim on both days 1 and 3, LM significantly increased myoblast proliferation in comparison with COL (*p* = 0.0017 and *p* = 0.0132 for days 1 and 3, respectively). These data suggest that LM is more conducive to myogenic cell survival and growth compared with COL during E-stim. No differences were observed in the nonstimulated groups, suggesting that both COL and LM support myoblast growth and viability to similar extents in the absence of stimulation. While the cell number on both collagen and LM substrates is similar in nonstimulated groups, cellular organization is different. On collagen, the cells aggregate to form clusters, while on LM, they are elongated and readily form myotubes, as shown in Figure 1.

### NO production

The total NO levels produced by myoblasts (Fig. 4D) showed a significant decrease on the COL substrate over 3 days in both unstimulated and stimulated groups. In contrast, NO levels showed a significant increase on the LM substrate over 3 days in both unstimulated and stimulated groups. Additionally, on day 3, NO levels were significantly higher on the LM substrate compared with COL in both unstimulated and electrically stimulated groups.

## Discussion

The goal of this study was to determine the effects of E-stim on C_2_C_12_ myoblasts cultured on either COL or LM substrate. Critical loss of muscle has been associated with fibrotic tissue deposition that primarily comprises COL. LM supplementation has been shown to dramatically improve the regenerative capacity of skeletal muscle in several models of muscular disease^11–13^ and injury^14^ by increasing satellite cell activity. Additionally, E-stim is undergoing investigation as a possible rehabilitation therapy targeted toward mimicking exercise.^19^

Higher expression of MyoD and myogenin was observed in electrically stimulated myoblasts cultured on both COL and LM on day 3 (Fig. 3B, C). MyoD and myogenin are crucial throughout the myogenesis process. MyoD is an early-stage marker of myogenic differentiation and is associated with myoblast proliferation, whereas myogenin is a late-stage marker of myogenic differentiation.^42,43^ Although no correlation was found between the substrate (COL or LM) and myogenic activity, these results show that E-stim itself supports myogenic activity. Multiple studies have demonstrated that E-stim causes cellular organization and growth. E-stim slows fiber formation and increases protein synthesis, mimicking *in vivo* effects of aerobic exercise.^44^ Studies have shown that E-stim can activate satellite cells,^45^ support myoblast fusion and myogenic differentiation,^45^ and increase MyoD expression.^46^ The findings from these previous studies further support results observed in this study that E-stim supports myogenic activity on day 3 of stimulation, irrespective of the substrate.

A previous study showed that mesenchymal stem cells cultured on COL decreased the expression of growth factors, cytokines, and ECM proteins in response to mechanical stimulation.^2^ A decrease in all of these components suggests that cellular interaction with COL decreases the cell's potential for myogenic repair and regeneration.^2^ Acutely, an increase in the proinflammatory marker IL-6 can signal satellite cell activation and initiate myogenesis^40,47^; however, chronically elevated levels of IL-6 have been associated with muscle wasting in cancer^40^ and aging.^48^ Our results show that electrically stimulated myotubes cultured on LM exhibited an increase in IL-6 on day 1, followed by a significant drop in IL-6 production on day 3, suggesting that E-stim activated myoblasts cultured on LM, but not COL. Furthermore, with continued E-stim, IL-6 production dropped to baseline levels at day 3, possibly to prevent cell damage (Fig. 4B). In support, studies have shown that IL-6 production tends to peak immediately following a short burst of exercise and then returns to baseline levels 2 days postexercise.^40^

E-stim of cells has the potential to stimulate mitochondrial biogenesis or myofibrillar protein synthesis, but further investigation is necessary to determine which method of exercise training is modeled by E-stim. Depending on the type of training, the muscle may respond by either upregulating mitochondrial biogenesis or myofibrillar protein synthesis. Mitochondrial biogenesis occurs as a result of endurance training; however, in this form of exercise, muscle growth does not occur.^49^ In mitochondrial biogenesis, pre-existing mitochondria grow and divide. Several studies have shown that physical activity increases mitochondrial content and those muscles that are continuously used have more mitochondrial activity than those used less frequently.^50,51^ Other studies have shown increases in mitochondrial enzymes and proteins after chronic E-stim in humans.^52–54^ SIRT1 is a major regulator of mitochondrial biogenesis during exercise.^55,56^ Activation of SIRT1 also leads to reduction of oxidative stress and inflammation, which ultimately results in suppression of fibrotic tissue deposition.^57^ Studies have shown that SIRT1 activity is correlated with a decrease in oxidative damage in mice with Duchenne muscular dystrophy.^58,59^

In this study, C_2_C_12_ myoblasts cultured on the LM substrate showed significantly higher SIRT1 expression and VEGF production (Figs. 3F and 4A) in response to E-stim. Similar to SIRT1, VEGF has been shown to protect against oxidative stress.^60^ SIRT1 is implicated in enhancing cellular growth, angiogenesis, and VEGF production.^61,62^ VEGF is a proregenerative growth factor that influences myoblast migration and survival.^38,39^ Satellite cells and regenerating myofibers highly express VEGF receptors. VEGF is capable of exerting strong antiapoptotic action on myoblasts, which promotes their survival and migration,^39,63^ and upregulation of VEGF is also seen in hypertrophic myofibers.^64^ Inhibition of VEGF is known to reduce differentiation and fusion of muscle-derived stem cells.^65^ In injured muscle, an increase in VEGF expression by myoblasts cultured on LM results in a proregenerative environment that stimulates both myogenesis and angiogenesis.^27^ Therefore, an increase in both SIRT1 production and VEGF expression in myoblasts cultured on LM may suggest that LM offers better protection from E-stim-mediated oxidative stress. However, more studies are needed to substantiate this claim. The XTT cellular proliferation assay showed a higher cell number on LM in response to E-stim on both days 1 and 3 compared with COL (Fig. 4C), which suggests that LM is better able to maintain cellular viability and minimize cell death in response to E-stim.

On day 1 of E-stim, significantly higher mTOR and atrogin-1 expression was observed in myotubes cultured on the LM substrate (Fig. 2G, H). mTOR, a serine/threonine kinase, senses environmental and intracellular cues and responds by influencing cell growth, differentiation, autophagy, survival, and metabolism.^66^ mTOR functions as a positive regulator of muscle hypertrophy and is vital for mitochondrial metabolism.^67^ Atrogin-1 can affect both muscle protein synthesis and degradation.^68,69^ We speculate that after the first bout of E-stim, myoblasts undergo higher levels of both protein synthesis and degradation on the LM substrate, resulting in higher expression of atrogin-1 and mTOR. Therefore, we postulate that electrically stimulated myotubes cultured on LM can better regulate protein metabolism.

NO production is associated with myoblast fusion.^70^ Previous studies have shown that E-stim can increase NO production in C2C12 cells *in vitro*. It has been reported that NO production regulates glucose influx and myoblast differentiation.^71^ NO also improves muscular strength and blood flow and activates mitochondrial biogenesis.^72^ Additionally, oxidative stress can reduce the production of NO and increase reactive oxygen species (O_2_^−^).^73^ In this study, NO production by myoblasts was significantly higher on the LM substrate in both stimulated and unstimulated groups on day 3 (Fig. 4D), further corroborating that LM offers several advantages to myogenic growth and activity compared with COL.

Overall, these results suggest that the LM substrate is more conducive to the myoblast growth and differentiation response to E-stim. While neither COL nor LM increased myogenesis, improved cell survival in response to E-stim was observed only in the presence of LM. On the LM substrate, myoblasts showed increased expression of markers associated with protein turnover and protection against oxidative stress. Based on results of this study, we propose that myoblast interaction with fibrotic tissue that primarily comprises COL may impair the beneficial effects of E-stim.

This preliminary study faced several limitations, the first being short time points. Future studies would extend the time points to evaluate the long-term effects of E-stim on myogenesis. Additionally, the metabolic markers studied were very limited. To expand this study and carry the implications into an *in vivo* model, an entire panel of metabolic markers would be investigated. Future work will include administration of LM into a mouse model of trauma or dystrophy before E-stim application to confirm the results of this study *in vivo*.

## Abbreviations Used

COL: collagen I
DMEM: Dulbecco's Modified Eagle Medium
ECM: extracellular matrix
E-stim: electrical stimulation
FFT: fast Fourier transform
GAPDH: glyceraldehyde 3-phosphate dehydrogenase
IL-6: interleukin-6
LM: laminin
mTOR: mammalian target of rapamycin
MyoD: myoblast determination protein 1
NO: nitric oxide
NS: no stimulation
PBS: phosphate-buffered saline
PDMS: polydimethylsiloxane
P/S: penicillin–streptomycin
SIRT1: Sirtuin 1
VEGF: vascular endothelial growth factor
